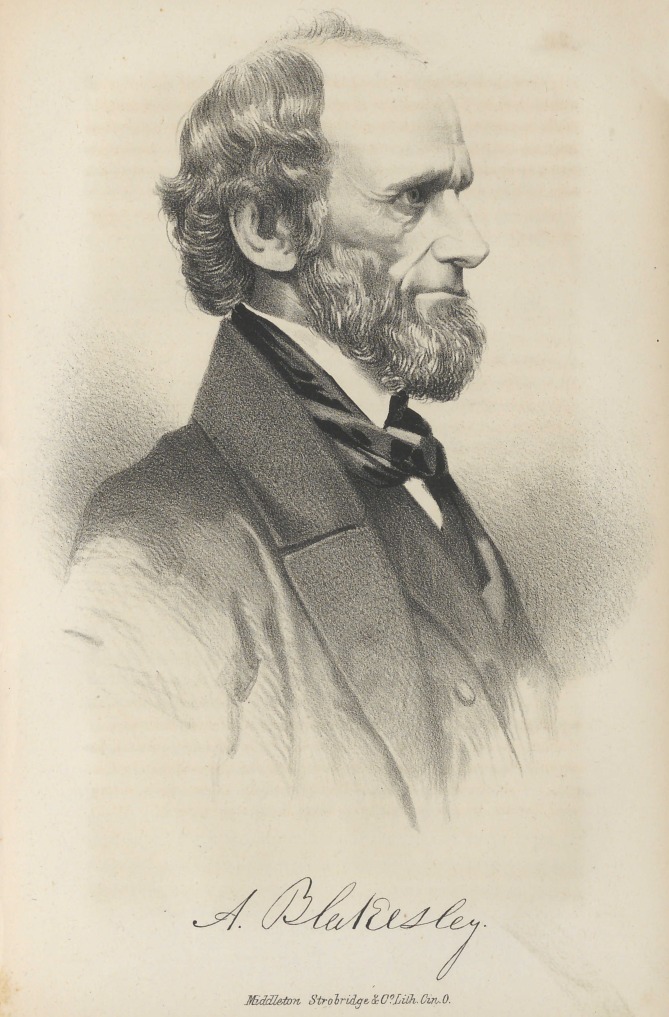# Editorial

**Published:** 1860-10

**Authors:** 


### Editorial.

### AMERICAN DENTAL ASSOCIATION.

This Society was organized, in Washington City, July 31st, 1860, by the adoption of the Constitution, published in our present issue. Upon the whole, the Association has made an encouraging start. Ten of the local and State Associations were fully or partially represented, and one of the Colleges. As a general rule, the new Societies were more promptly represented than the old. The Ohio Dental College Association, entitled to a large delegation, had no representative, while the Mississippi Valley Association had but one. The St. Louis Society, and the Faculty of the Ohio College, were also unrepresented. We are prepared to say, that the sparseness of Western delegates is to be attributed more to the place of meeting, than to a want of interest. The next meeting will be more nearly central ; and the result will be different. The Faculty of the Baltimore College was not represented, nor were the New York State, and New York City Societies. The City Society, as explained elsewhere, appears to be unfriendly to the movement. Why the others were not on hand, we are not able to say.

It can not be expected that a meeting for organization would be very interesting, or important to science, in its immediate results ; yet this constitutes at least a partial exception to the rule. To vary the exercises, some five essayists were appointed, at the preliminary meeting at Niagara, but one of whom responded. The rest of us ingloriously failed ; but, fortunately, several volunteers came to the rescue, and they, accordingly, have the thanks of at least one who was too negligent to respond to his appointment.

We stated above, that "the rest of us ingloriously failed ;" but this may not be strictly true. From the *Cosmos* we learn that "Dr. McQuillen presented an oral synopsis of a paper upon The Anatomy, Physiology, Pathology, and Remedial Treatment of the Fifth Pair of Nerves." The papers *read* having been mentioned, in order, just previous to this, we are further told that, "On motion, the above papers were requested for publication. Copies being directed to be placed in the hands of the Committee of Publication." From the New York Journal we learn the same in regard to the *synopsis*, and further, that "Dr. Barker moved that the paper be referred to the Committee of Publication; which was agreed to. Subsequently, however, Dr. McQuillen declined furnishing the essay, as he wished to publish it in the Cosmos."

According to a natural inference from the Cosmos, there were but three failures among the appointed essayists. According to the Journal, there were three failures and a ——. Dr. M. had an undoubted right to publish his article in the Cosmos; but why tease the Association with it? A boy would be considered boorish, who would tell his schoolmates that his satchel was full of sugar plums, but they shouldn't have any.

The Constitution, as adopted, is a good one. The original report was much improved by amendments. Even our correspondent, "E. T." will hardly complain. He should not, at any rate; for all his suggestions, but two or three, were carried out.

We expect great good from the Association. Its present Standing Committees are composed of working men. And when it comes to be understood that the annihilation of the "American Convention" is no part of its programme, the Association will, no doubt, count, among its friends, many who now stand aloof, through fear or jealousy of its influence on the former body.

We hope that local and State Societies will be organized all over the land, and that they will all be fully represented at Cleveland next July. W.

### THE MALLET—RUBBER WORK.

THE question is often asked, "do you use the mallet for filling teeth yet, and are you as much pleased with it now as at first?" to which we reply we do, and are still better pleased with it, the longer we use it. It is applicable in almost all cases; and there is not more than one case in twenty, in which we do not use it. Patients who have become accustomed to it, will not have fillings condensed in any other way. The great points are, the efficiency, and the ease, to the operator, with which the work is done.

We can now operate ten hours, with less fatigue than we could six, with hand pressure, and make more satisfactory work. None who properly test it, will give it up.

RUBBER WORK.—The question is also asked almost every day, "What about rubber work?" We have at different times expressed our opinion; but the desire is to know what developments time is making in regard to it. All those who are using the vulcanite process, have equal opportunities of testing its efficiency. In regard to durability, there are no new developments, that we know of. We have seen some cases which proved failures, after being worn for a while in the mouth; for instance, two or three pieces, after being worn a few months, became so friable, as to break as readily as a piece of thin porcelain; in other cases they were porous, and absorbed the fluids of the mouth, and became intolerably offensive, so that they had to be dispensed with. These may be difficulties that are dependent upon the manipulation, or upon the material; there is some of the rubber prepared by the Rubber Company, of a very inferior quality; whether it depends upon the imperfect preparation of the rubber, or a defect in the materials primarily, we do not know; but it would be very desirable to have a uniformly good article.

In regard to making the work, we think there is no economy, either in time, skill or labor, over other styles, if justice is done to the work. T.

### OHIO COLLEGE OF DENTAL SURGERY.

THE regular session of this institution will commence on the first Monday of November, and continue till the 20th of February next.

The arrangements are such as to give the most thorough instruction in each department. Arrangements have been made for illustrations and demonstrations with the microscope; a very fine instrument is at the disposal of the College, and the assistance of a good practical microscopist has been secured. There is a very large collection of anatomical, physiological and pathological preparations in the College; there is, indeed, everything in this way that could be desired. It is very important that all who design to attend should be in as early as possible. T.

### THE AMERICAN DENTAL CONVENTION.

This body has gone East. Indeed, it always had an inclination to do so. Its grandfather, the old American Society, came West once, and barely got home in time to die. And when he was all ready to die, he couldn't do it, till some of his western sons went and helped him to "kick the bucket." The Convention came West once. Not far West, either ; but it was so frightened that it sped back, stopped for breath at Niagara, hastened on to Saratoga, took a drink of Congress water, and is now on its way to New Haven, where we hope it will so far recover from its western fright as to venture a short distance from the eastern shore.

When the Convention met in Cincinnati, there were enough of western men present to have taken it, the next year, to St. Louis or Chicago. And if they had done so, there would have been a still greater preponderance of western members ; and they could have taken it to Leavenworth, and the next year, to Denver City. But our western brethren thought such a course would not be generous ; and they didn't do it. How much farther east are you going to take it, brethren ?

Personally, however, we care but little where it is ; but many worthy dentists in the West would like to attend its meetings ; but, while it is confined to the Atlantic shore, it is impracticable for most of them to do it. It was never expected that it would meet in the West as often as in the East ; but we think that one meeting in seven is hardly a fair divide. Why could it not meet in more accessible and central localities ? W.

---

### PASSAGE OF SERUM THROUGH DENTINE.

In an editorial in the last number of the Register, a suggestion of Dr. Allport is referred to, which is liable to be misunderstood. After the opinion that dentine may be easily prepared, so as to preclude the passage of any fluid through it, it is stated,—"This may be done by the application of creosote, or any similar oil, applied to the dentine, which it absorbs, till it becomes saturated, and this prevents the passage of any other fluid."

Now, the mere absorption of creosote, to complete saturation, would not prevent the passage of another fluid, any more effectually than moistening with stagnant water would stop the leaks of a mill-dam. But when the chemical action of creosote is considered, in connection with its absorption, the force of the suggestion may be appreciated. Creosote is an acid, and forms insoluble, non-putrescent compounds with albuminous substances generally; and in this way it acts, in excluding other fluids. This is, evidently, Dr. Allport's idea. It is the one we have always maintained, and one to which we have frequently alluded. W.

### "DUTIES OF CHEMISTS."

UNDER the above title, "A. B.," of the American Dental Review, retreats from the bold position taken in the preceding number. He *Review*-ed it, and found it untenable. In answer to the charge of "designedly" misquoting the language of "our comrade," he attempts to make merry over his office arrangements.

But he wishes to be excused for "want of experience;" and we must excuse him. In fact, we do excuse him. But we hope it will not take a long experience to enable him to copy a man's remarks when they are in print.

After stating that his demand of "the professor of chemistry" was made with all due respect, which we believed all the time, he says: "But what we do dislike is, that Professors, when commenting on articles which are offered for practical use to the professional world, do not call them by their proper names, such as they would, most assuredly, give them, were they about to expatiate on the merits of the article before their class in College."

This is all very well. But if a sensible professor were about to expatiate on the merits of the article under consideration, he would, "most assuredly," call it "Roberts' Os-artificial," just as he would call a well known liquid, water, and not protoxyd of hydrogen. That the composition of the article was, in the main, pretty well known, was evidenced by A. B.'s familiarity with it. W.

## Figures and Tables

**Figure f1:**